# Baseline predictors of cognitive change in the treatment of major depressive episode: systematic review

**DOI:** 10.1192/bjo.2020.114

**Published:** 2020-10-30

**Authors:** Zoe A. Barczyk, Katie M. Douglas, Richard J. Porter

**Affiliations:** Department of Psychological Medicine, University of Otago, New Zealand; Department of Psychological Medicine, University of Otago, New Zealand; Department of Psychological Medicine, University of Otago; and Clinical Research Unit, Canterbury District Health Board, New Zealand

**Keywords:** Depressive disorders, bipolar affective disorders, out-patient treatment, psychological testing, in-patient treatment

## Abstract

**Background:**

Cognitive impairment is a core feature of depression and has a negative effect on a person's functioning, in psychosocial and interpersonal areas, and on workforce performance. Cognitive impairment often persists, even with the remittance of mood symptoms. One potential way of improving treatment of cognitive impairment would be to identify variables that predict cognitive change in patients with depression.

**Aims:**

To systematically examine findings from studies that investigate baseline variables and how they predict, or correlate with, cognitive change in mood disorders, and to examine methodological issues from these studies.

**Method:**

Studies that directly measured associations between at least one baseline variable and change in cognitive outcome in patients with current major depressive episode were identified using PubMed and Web of Science databases. Narrative review technique was used because of the heterogeneity of patient samples, outcome measures and study procedures. The review was registered on PROSPERO with registration number CRD42020150975.

**Results:**

Twenty-four studies met the inclusion criteria. Evidence from the present review for prediction of cognitive change from baseline variables was limited for demographic factors, with some preliminary evidence for depression, cognitive and biological factors. Identification of patterns across studies was difficult because of methodological variability across studies.

**Conclusions:**

Findings from the present review suggest there may be some baseline variables that are useful in predicting cognitive change in mood disorders. This is an area warranting further research focus.

Major depressive disorder (MDD) is prevalent worldwide, with estimates of 4.4% of the population affected in 2015.^1^ It is one of the leading causes of disability internationally.^2^ Cognitive impairment is now widely recognised as a core symptom of depression,^3–5^ within a MDD or bipolar disorder presentation, and also in euthymia.^6^

Cognitive domains affected in depression include executive function, attention/concentration, learning/memory and processing speed, as well as so called ‘hot’ areas of cognition, which involve emotion processing.^7^ Cognitive impairment often persists, even with resolution of mood symptoms.^6,8^ Cognitive impairment has a negative effect on a person's psychosocial and interpersonal functioning, and on performance in the workforce.^9,10^ Preliminary evidence also suggests that poor cognitive functioning increases the rate of relapse for depressive disorders.^11,12^

Given the effect on patients, and the fact that current treatment does not adequately address cognitive dysfunction, cognitive impairment has been identified as an important feature of depression, and a clear target in treatment and research.^6,7,13^ One potential way of improving treatment of cognitive impairment would be to identify variables that predict cognitive change in patients with depression. This identification could help target treatment or identify those who may need extra intervention. It would also identify a group in whom research is particularly needed to address this problem. There is particular clinical significance if predictors are identified that are relatively quick and easy to measure in clinical practice.

A further issue is that, particularly in the elderly, depression may be associated with a greater decline in cognitive function over time than that seen in healthy aging.^14^ However, the underlying aetiology of this decline, and what particular aspects of the presentation of depression predict decline, is not clear. Understanding and being able to predict which factors predict longer-term cognitive decline in patients with depression is desirable to utilise and develop treatments that mitigate this.

Two scenarios are present in the literature: studies examining predictors of cognitive improvement (usually short-term treatment trials) and studies examining predictors of worsening cognitive function (usually longer-term studies in older participants).

A recent systematic review has been conducted to examine whether cognitive function predicts mood outcomes in treatment studies of depression;^15^ however, to date, to our knowledge, there has been no systematic review to investigate predictors of cognitive change related to treatment in mood disorders.

## Aims

The aims of the present review were therefore as follows: to systematically examine findings from studies that investigate variables that predict or correlate with cognitive change in mood disorders, and to examine methodological issues from studies examining these variables.

## Research question

Are there baseline predictors of cognitive change in patients with mood disorders?

## Method

### Protocol and registration

Details of the protocol for the current systematic review were registered on PROSPERO with registration number CRD42020150975. No ethics approval was required, as this is a review using existing studies only.

### Search strategy

Up to 29 August 2019, electronic database searches were conducted using PubMed. Search terms used in the initial search were a combination of ‘cognitive’ *or* ‘neuropsychological’ *and* ‘function’ *and* ‘change’ *or* ‘improvement’ *and/or* ‘treatment’ *and* ‘depression’ *or* ‘bipolar’. Reference lists of relevant papers found were reviewed to find additional papers. Web of Science was used to access articles that cited the relevant articles found through the mentioned methods, to obtain more recent studies. Searches were also conducted in EMBASE and PsycINFO, but no additional studies were identified.

### Inclusion criteria

Articles were selected on the basis that they included a sample who were in a current major depressive episode (as part of MDD or bipolar disorder) at baseline and included individuals over the age of 18 years. Studies needed to include assessment of cognitive functioning at baseline, as well as measurement of cognitive functioning after treatment or at the end of follow-up, in the case of naturalistic studies. To answer the research question, studies were required to directly measure associations between change in cognitive outcome and at least one baseline variable. Studies were able to use different methodologies to assess associations, for example, correlation or regression. Type of treatment used was not restricted for inclusion, with the exception of electroconvulsive therapy, which was excluded. For example, pharmacotherapy, cognitive training, repetitive transcranial magnetic stimulation (rTMS), standard hospital care or environmental therapies such as exercise were all valid for inclusion. Naturalistic studies in which no specific treatment was being investigated were also included.

### Exclusion criteria

Reasons for exclusion were (i) the use of a sample with comorbid major medical, neurological or endocrinological conditions; (ii) the predictor variable was investigated in relation to changes in depression, but not cognitive functioning; (iii) a predictor or correlate that was measured at a point other than baseline (e.g. change in depression severity score) and (iv) the sample was euthymic at the time of baseline assessment. Only English language articles were included.

### Full study review

Initially, one reviewer screened articles, reviewing the titles and abstracts to determine whether the full text should be reviewed. The same reviewer then read the full texts to determine whether they met the inclusion criteria. If it was unclear whether a study met the inclusion criteria, this was discussed with a second co-author, and then a third co-author if further clarification was required. Data was obtained from eligible studies and collated in a spreadsheet. The following information was extracted: sample characteristics, including sample size, gender and age; study design; mood disorder measurements; cognitive tests used; baseline predictors of cognitive change; and study outcomes. The applicable Joanna Briggs Institute checklists were used as a formal risk-of-bias tool for each study, and checklists are available from authors on request. Differences in design and assessment between studies were also discussed between co-authors, and the quality of each study taken into account when synthesising the evidence.

## Results

### Study characteristics

**Fig. 1:**
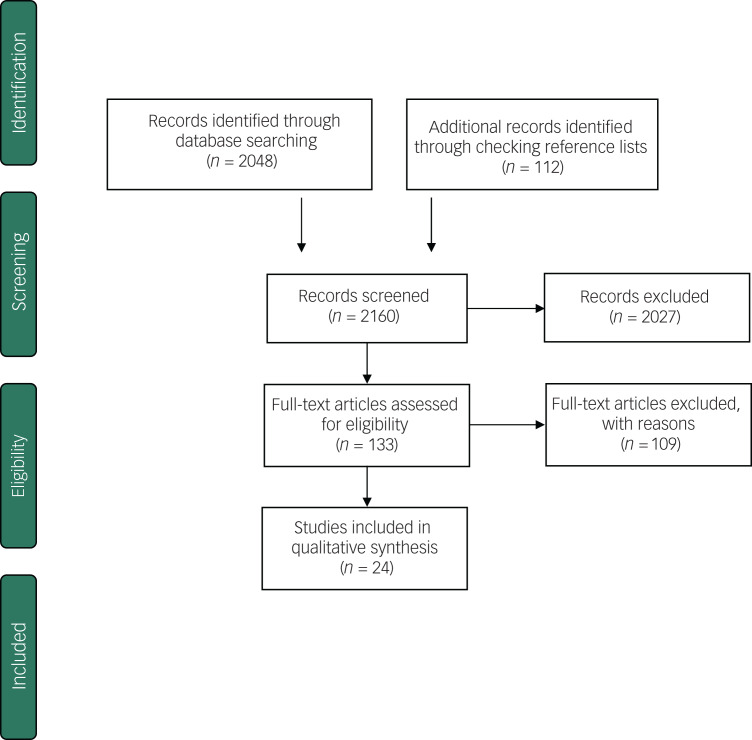
Preferred Reporting Items for Systematic Reviews and Meta-Analyses flow diagram.

Twenty-four studies met the inclusion criteria (total *n* = 3403), Fig. 1 shows the Preferred Reporting Items for Systematic Reviews and Meta-Analyses (PRISMA) flow diagram of studies retrieved for this review. Of the 24 studies, 12 involved participants aged between 18 and 64 years old, referred to in this review as adult samples. In 12 studies, participants recruited were 65 years or older, referred to as older adult samples (see Tables 1 and 2).

**Table 1:** Adult sample studies included in review

Reference	*N*	Mean age, s.d.	Study design	Treatment	Depression severity measurement	Cognitive outcome measures	Predictors tested
Hoffman et al^16^	202 out-patients, MDD	51.7 ± 7.6	4-month randomised controlled trial	Physical activity: supervised or home-based	BDI ≥12	TMT, Stroop, Ruff 2 & 7 Test, DSST, Logical Memory, Verbal Paired Associate, Animal Naming, Controlled Oral Word Association Test, Digit Span	Age, education, baseline cognitive performance
Jeon et al^17^	164 out-patients, MDD	58.4 ± 10.8 tianeptine, 57.1 ± 9.8 escitalopram	12-week randomised open-label trial	Tianeptine, escitalopram	HRSD-17 ≥ 16	Continuous Performance Test, Verbal Learning Test, Raven Progressive Matrices	Age, gender, education, baseline cognitive performance
Lin et al^18^	353 in-patients, MDD	34.9 ± 12.9	6-week prospective semi-naturalistic open-label longitudinal study	Antidepressants, psychiatrists prescribed type and dosage as saw fit	Psychiatrist diagnosis, HRSD-17 to measure change	TMT, Digit Symbol Coding, Digit Span, Modified Wisconsin Card Sorting Test, Tower of Hanoi, Animal Naming, Immediate Visual Reproduction	Depression subtype*
Nadeau et al^19^	48 participants, medically resistant major depression >6 years	46.6 ± 14.2	2-week randomised controlled trial, 3-month follow-up	Repetitive transcranial magnetic stimulation	HRSD-24 ≥ 18 (with ≥3 on item 1)	TMT, Stroop, Boston Naming Test, category fluency, Block Design, Controlled Oral Word Association Test, California Verbal Learning Test, Paced Auditory Serial Addition Test	Age, gender, baseline depression severity, baseline cognitive performance*, handedness
Soczynska et al^20^	41 out-patients, MDD	34.6 ± 9.9 bupropion, 41.3 ± 12.9 escitalopram	8-week double-blind randomised controlled comparative trial	Bupropion XL, escitalopram	HRSD-17 ≥ 16	California Verbal Learning Test, Logical Memory, Digit Span, Spatial Span, Letter-Number Sequencing, Brief Visuospatial Memory Test-Revised	Education*
Mikoteit et al^21^	25 patients, MDD	42.9 ± 12.3	6-week open-label prospective clinical study	Duloxetine	ICD-10, HRSD-17 ≥ 17	‘Test zur Aufmerksamkeitsprüfung’ (Test Battery for Attentional Performance)	Baseline depression severity, baseline cognitive performance*
Miskowiak et al^22^	84 patients, bipolar disorder in partial remission or current unipolar depression	40 ± 10 erythropoietin, 43 ± 12 saline	8-week randomised controlled trial	Erythropoietin	Unipolar depression: HRDS-17 ≥17; bipolar disorder partial remission: HRSD-17 ≤ 14, YMRS ≤14	Rey Auditory Verbal Learning Test	Age, gender, diagnosis
Miskowiak et al^23^	79 patients, bipolar disorder in partial remission, or current unipolar depression	No mean provided; 20–35 years: 12; 35–50 years: 18; 50–70 years 9	8-week randomised controlled trial	Erythropoietin	Unipolar depression: HRDS-17 ≥17; bipolar disorder partial remission: HRSD-17 ≤ 14, YMRS ≤14	Rey Auditory Verbal Learning Test	Age, gender, education, longer illness duration*, diagnosis, baseline depression severity, number of mood episodes, objective memory dysfunction baseline*, subjective cognitive complaints*
Ott et al^24^	79 patients, bipolar disorder in partial remission, or current unipolar depression	41.2 ± 10.8 erythropoietin, 42.6 ± 12.3 saline	8-week randomised controlled trial	Erythropoietin	Unipolar depression: HRDS-17 ≥17; bipolar disorder partial remission: HRSD-17 ≤ 14, YMRS ≤14	Rey Auditory Verbal Learning Test, RBANS, Verbal Fluency, WAIS-III Letter-Number Sequencing, TMT, Rapid Visual Information Processing, Massachusetts General Hospital Cognitive and Physical Functioning Questionnaire	Baseline objective cognitive deficits*, subjective cognitive complaints
Thomas et al^25^	77 participants, 46 with severe mood disorders, 31 with schizophrenia or schizoaffective disorder	44.43 ± 11.27	12-week randomised controlled trial	Compensatory cognitive training	DSM-IV	TMT-A, TMT-B, BACS, Symbol Coding, Category Fluency, Continuous Performance Test-Identical Pairs, WAIS-III Digit Span, WMS-III Spatial Span, University of Maryland Letter-Number Span, HVLT-R, Brief Visual Memory Test-Revised, Neuropsychological Assessment Battery Mazes, Wisconsin Card Sorting Test, Letter Fluency, Memory for Intentions Screening Test	Age*
Lee et al^26^	86 patients, MDD	48.8 ± 11.1	12 weeks, observational	Antidepressant monotherapy	DSM-V diagnosis	Perceived Deficits Questionnaire-Korean version	Age, gender, education, occupation, MADRS score*, SDS, EQ-5D*, sick days before entry*
Buschert et al^27^	43 in-patients, unipolar depression	47.27 ± 6.84 experimental, 47.47 ± 8.47 controls	4-week randomised controlled trial	Physical activity: endurance training	ICD-10, BDI, HRSD-7	‘Test zur Aufmerksamkeitsprüfung’ (Test Battery for Attentional Performance)	Education, baseline performance

MDD, major depressive disorder; BDI, Beck Depression Inventory; TMT, Trail Making Test; DSST, Digit Symbol Substitution Test; HRSD, Hamilton Rating Scale for Depression; YMRS, Young Mania Rating Scale; RBANS, Repeatable Battery for the Assessment of Neuropsychological Status; BACS, The Brief Assessment of Cognition in Schizophrenia; WAIS-III, Wechsler Adult Intelligence Scale; WMS-III, The Wechsler Memory Scale, 3rd edition; HVLT-R, Hopkins Verbal Learning Test-Revised; MADRS, Montgomery–Åsberg Depression Rating Scale; SDS, Sheehan Disability Scale; EQ-5D, EuroQol-5 dimensions questionnaire.

* *P*<0.05.

**Table 2:** Older adult sample studies included in review

Reference	*N*	Mean age, s.d.	Study design	Treatment	Depression severity measurement	Cognitive tests	Predictors tested
Shorter studies (≤4 months)							
Devanand et al^28^	39 out-patients; MDD, dysthymic or depression NOS	72.0 ± 10.2	12-week open-label trial	Sertraline	HRSD-17 ≥ 8	DSST, Boston Naming Task, SRT, Animal Naming & CFL Verbal Fluency, Selective Reminding Task, Letter & Shape Cancellation Tasks	Age, education
Doraiswamy et al^29^	444 out-patients, MDD	68.0 ± 5.7	12-week double-blind studies (pooled from 2)	Sertraline, fluoxetine, nortriptyline	HRSD-24 ≥ 18	MMSE, DSST, Shopping List Task	Age, depression severity, baseline cognitive impairment
Nebes et al^30^	73 in- and out-patients, MDD	70.3 ± 6.4 depression, 71.0 ± 7.2 controls	12-week randomised controlled trial	Nortriptyline or paroxetine	HRSD-17 ≥ 15	MMSE, Dementia Rating Scale, DSST, N-Back Test, TMT, Rey Auditory Verbal Learning Test	Age at onset, baseline cognitive functioning
Raskin et al^31^	311 patients, MDD	72.6 ± 5.7 duloxetine, 73.3 ± 5.7 placebo	8-week randomised controlled trial	Duloxetine	HRSD-17 ≥ 18	Rey Auditory Verbal Learning Test, DSST, Two-Digit Cancellation Test, Letter-Number Sequencing Test	Age; gender; ethnicity; number of previous drugs/episodes; GDS total score; CGI severity scale score; HRSD anxiety and insomnia; comorbid arthritis, diabetes, vascular disease or any of these; or mild dementia
Han et al^32^	281 in-patients with major, minor, and without depression	78.7 ± 7.1 major, 78.4 ± 6.9 minor, 79.9 ± 7.3 without	12-month follow-up quasi experimental trial	Antidepressant treatment as prescribed	DSM-IV criteria	MMSE	Depression severity*
Barch et al^33^	166 out-patients, MDD	68.1 ± 7.1	12-week open-label trial	Sertraline	SCID	TMT, Wisconsin Card Sorting Test, DSST, Boston Naming Task, Benton Visual Reminding Test, Stroop, Mattis, World List Learning, Digit Span, Rey–Osterrieth Complex Figure Test, Shipley Vocabulary Test	Age*, baseline depression severity*, age at onset*, baseline cognitive function*, vascular risk score*, white matter hyperintensities*
Marano et al^34^	17 patients with MDE, 17 controls	66.9 ± 6.4 depression, 66.0 ± 7.9 comparison	12-week open-label trial	Citalopram	HRSD-17 ≥ 15	California Verbal Learning Test, Delis–Kaplan Executive Function System	Grey matter volumes*
Pelton et al^35^	18 out-patients, MDD or dysthymic disorder	65.3 ± 8.9	12-week placebo-controlled pilot trial	Donepezil	HRSD-24 ≥ 14	MMSE, Selective Reminding Task, DSST, TMT, CFL Verbal Fluency	Olfactory performance*
Longer studies (>4 months)							
Lohman et al^36^	1401 participants, 310 with elevated depression symptoms	73.5 ± 6.0 memory training, 74.0 ± 6.5 controls	5-year follow-up, 6-week randomised controlled trial	ACTIVE memory training	CES-D-12 ≥ 9	Rey Auditory Verbal Learning Test, Hopkins Verbal Learning Test	Baseline depression severity*
Parisi et al^37^	1375 participants, 322 with elevated depression symptoms	74.1 ± 6.0	10-year follow-up, 6-week randomised controlled trial	ACTIVE reasoning training	CES-D-12 ≥ 9	Word Series, Letter Series, Letter Sets	Baseline depression severity
Pelton et al^38^	35 patients with MDD, dysthymic disorder or depression NOS	66.5 ± 9.8 donzepil, 67.0 ± 9.0 placebo	48-week open-label pilot study	Escitalopram, memantine	HRSD-24 > 13	Selective Reminding Task, MMSE, Boston Naming Task, TMT, DSST, Benton Visual Reminding Test, CFL Verbal Fluency, Wechsler Memory Scale Visual Reproduction	Age, gender, education, baseline depression severity
Olaya et al^39^	1027 total participants, 291 with past or present depression	70.5 ± 7.3	3-year naturalistic follow-up	Naturalistic	CIDI, algorithm based on DSM-IV criteria	Word List Learning, Animal Naming Task	Age at onset

MDD, major depressive disorder; NOS, not otherwise specified; HRSD, Hamilton Rating Scale for Depression; DSST, Digit Symbol Substitution Test; SRT, Selective Reminding Test; CFL, the 3 letters used in this particular verbal fluency test; MMSE, Mini-Mental State Examination; TMT, Trail Making Test; GDS, Geriatric Depression Scale; CGI, Clinical Global Impression; SCID, Structured Clinical Interview for DSM; MDE, Major Depressive Episode; ACTIVE, Advanced Cognitive Training for Independent and Vital Elderly; CES-D-12, Center for Epidemiologic Studies Depression Scale; CIDI, Composite International Diagnostic Interview.

* *P* < 0.05.

Overall, the quality of studies as measured with the Joanna Briggs Institute checklists was good. Across the randomised controlled trials (RCTs), there were several criteria that were most commonly not clearly stated in the article if they were met: whether the studies used true randomisation, whether allocation to treatment groups was concealed and whether outcome assessors were blind to treatment assignment. These studies were still included, as they otherwise met checklist criteria, and these areas were unclear rather than confirmed not met; for example, stating participants were randomised to conditions, but not clarifying how this took place.

Studies used a range of measures of cognitive function. This included one study with a subjective measure only,^26^ whereas the majority used objective cognitive measures. Three studies used one cognitive test,^22,23,26^ whereas the remaining 21 studies used multiple tests. For mood disorder measures, 16 studies used the Hamilton Rating Scale for Depression (HRSD), although criteria for entry ranged from a HRSD score of ≥8 to a score of ≥18. To attempt to separate studies into those that followed only a treatment course compared with those that followed for a longer period, studies were divided into ‘shorter’ and ‘longer’ studies. The studies naturally split into those ≤4 months (shorter studies), and those ≥48 weeks (longer studies). There were 19 shorter studies and 5 longer studies.

#### Adult samples

Of the adult samples, there were 12 shorter studies, across 10 samples (total *n* = 1123). There were no adult sample longer studies. Ott et al,^24^ Miskowiak et al^22^ and Miskowiak et al^23^ are three secondary data analysis papers that used the same sample, but investigated different outcomes.

Regarding treatment type, within adult samples, six studies used antidepressant treatment. Another study used standard hospital care as the treatment, so specifics varied between patients but involved medication prescribed by psychiatrists, as appropriate.^18^ Two studies used physical activity as the treatment.^16,27^ For one study, the treatment was rTMS,^19^ and the final study used compensatory cognitive training as the treatment.^25^

#### Older adult samples

Of the 12 older adult studies (total *n* = 2307), five were longer and seven were shorter studies. A total of 9 out of 12 studies used antidepressant treatment for the intervention. Two older adult studies were based on two different training groups from the Advanced Cognitive Training for Independent and Vital Elderly trial.^36,37^ These used memory^36^ and reasoning^37^ training as treatment. The remaining older adult study was a 3-year, naturalistic follow-up study.^39^

The following sections are divided by predictor type and will outline the adult sample studies first, followed by the older adult samples. It was expected that over the short-term follow-up studies, while depression was being treated, there would be an improvement in cognitive function, whereas in the longer-term follow-up studies, there would be a decrement in cognitive function. As such, within these sections, studies are discussed by short- and long-term follow-up.

### Demographic factors

#### Age

##### Adult samples

Seven adult sample studies (total *n* = 661) examined age as a variable related to cognitive change. One shorter study^25^ reported mixed significant results.

Thomas et al^25^ conducted a 12-week RCT for compensatory cognitive training (*n* = 77). Age was a significant moderator for category fluency, for which younger participants showed more of an improvement over time. However, results were not analysed separately for the two broad diagnostic groups of severe mood disorder (*n* = 46) and schizophrenia or schizoaffective disorder (*n* = 31), so the effect of age on cognitive change for patients with mood disorder alone cannot be commented on.

The remaining six studies (total *n* = 584) found no significant relationship between age and cognitive change.^16,17,19,22,23,26^ Jeon et al (*n* = 164)^17^ included age in an analysis, but did not report finding a significant relationship. It is assumed that age did not significantly affect cognitive change.

##### Older adult samples

Four shorter studies (total *n* = 960) examined age as a predictor of cognitive change in older adult samples.

One shorter study^33^ (*n* = 166) found mixed results depending on cognitive area. Barch et al^33^ measured cognitive functioning before and after a 12-week course of sertraline in adults aged 60 years and older with MDD. Older age was associated with less improvement for the areas of working memory (*r* = −0.25, *P* < 0.005), executive function (*r* = −0.17, *P* < 0.05) and psychomotor speed (*r* = −0.18, *P* < 0.05). No significant relationship for language function or episodic memory was found.

The three remaining studies (total *n* = 794) found no significant relationship between age and cognitive change.^28,29,31^

##### Longer follow-up studies

Pelton et al (*n* = 35)^38^ examined age as a predictor of cognitive change over 48 weeks in their older adult study, with non-significant results.

##### Age as a predictor of cognitive change

Only one adult study and one older adult study found significant results, despite 11 studies examining age. Thomas et al^25^ had a sample that was mixed between patients with mood disorders and patients with schizophrenia, so it is possible this influenced results. They also found the significant result for one domain only, despite running multiple cognitive tests. The combined sample size of the studies with significant results was also small compared with the combined negative studies. Thus, there is limited evidence from the studies identified that age is a predictor of cognitive change. A confounding methodological issue is, however, that none of the studies were conducted over an extended age range.

#### Gender

##### Adult samples

Gender in relation to cognitive change was investigated in five shorter studies (total *n* = 382). None of these studies found a significant relationship between gender and cognitive change.^17,19,22,23,26^

##### Older adult samples

In the two studies examining it (total *n* = 755),^29,31^ gender was not found to have a significant relationship with cognitive change.

##### Longer follow-up studies

Pelton et al (*n* = 35)^38^ examined gender as a predictor of cognitive change over 48 weeks in older adults, with non-significant results.

#### Education

##### Adult samples

One of six shorter studies (total *n* = 615) that examined education as a predictor of cognitive change showed significant results. In an RCT of bupropion and escitalopram for patients with MDD (*n* = 41), Soczynska et al^20^ reported that for immediate verbal memory, higher level of education was correlated with greater improvement. No significant relationship between education and cognitive change was found in the remaining studies (total *n* = 574).^6,16,17,23,27^

##### Older adult samples

The one older adult study (*n* = 39) that examined education found no significant relationship with cognitive change.^28^

##### Longer follow-up studies

Pelton et al (*n* = 35)^38^ examined education as a predictor of cognitive change in their 48-week, older adult study, with non-significant results.

#### Ethnicity

##### Older adult samples

Raskin et al (*n* = 311)^31^ reported no significant interaction between ethnicity and treatment for change in composite cognitive score.

#### Handedness

##### Adult samples

Nadeau et al^19^ investigated handedness in their shorter study and found no significant relationship with cognitive change in the areas of language, visuospatial function, executive function, verbal episodic memory and attention.

### Depression factors

#### Baseline depression severity

##### Adult samples

Depression severity as a predictor of cognitive change was investigated by four studies (total *n* = 238).^19,21,23,26^ Lee et al (*n* = 86)^26^ found a significant relationship between depression severity and the subjective Perceived Deficits Questionnaire-Korean version (PDQ-K), for total score as well as in the domains of attention/concentration and organisation/planning. For these areas, greater baseline depression severity was associated with greater subjective cognitive improvement. A significant relationship for prospective memory was not found.^26^

The three remaining studies (*n* = 152) found no significant relationship between depression severity at baseline and cognitive change.^19,21,23^

##### Older adult samples

Three shorter studies (total *n* = 921) examined baseline depression severity in association with cognitive change, with mixed results.

Barch et al^33^ found higher baseline Montgomery–Åsberg Depression Rating Scale score was correlated with less improvement in visual episodic memory (*r* = −0.16, *P* < 0.05); however, they had non-significant results for working memory, executive function, language function or psychomotor speed in their 12-week, open-label trial (*n* = 166). The remaining two studies found no significant relationship between depression severity at baseline and cognitive change.^29,31^

##### Longer follow-up studies

Four longer studies (total *n* = 948) examined baseline depression severity in association with cognitive change, with mixed results.

Han et al^32^ assessed 281 in-patients aged 65 years and older over 12 months. Participants were taking antidepressant medication, and were pooled from a RCT and an observational cohort study. Han et al analysed groups separately based on major, minor or no depression, according to DSM-IV criteria. Only the group with minor depression showed a significant improvement on the Mini-Mental State Examination (MMSE) during their trial.

Lohman et al^36^ examined the relationship between depressive symptoms and memory performance with training, using participants from a memory training group and a control group (*n* = 310) followed up over 5 years. Participants were community-dwelling adults aged over 65 years. A significant association between elevated depressive symptoms, measured with the 12-item version of the Centre for Epidemiological Studies-Depression Scale (CESD-12), and change in recall and recognition memory was reported, indicating those with elevated depressive symptoms experienced a faster decline in memory. Parisi et al^37^ examined participants from the reasoning training arm of the same study (*n* = 322), at the 10-year follow-up. CESD-12 scores at baseline were not significantly associated with change in reasoning performance, which was the only domain assessed.

Pelton et al (*n* = 35)^38^ found no significant correlation between baseline HRSD scores and changes on cognitive measures.

##### Depression severity as a predictor of cognitive change

There is conflicting evidence between age groups. Among the adult samples, the only study to find significant results for severity used a subjective measure of cognitive functioning as the outcome measure.^26^ The measure being subjective may have some issues, and the authors acknowledge the lack of objective tests as a limitation, although they do note that their measure (PDQ-K) has been reported to significantly correlate with objective measures in the literature.^26,40^ It might be expected that greater depression severity is associated with greater cognitive impairment and therefore greater chance of improvement with treatment. This may in fact underlie the significant result in Lee et al^26^ However, it may also be that greater depression severity in the elderly is associated with greater neurobiological disturbance, which may be irreversible. For example, vascular changes may be associated with more severe depression,^41^ which, as discussed below, appears to be associated with less cognitive improvement in response to treatment.^33^

There was some evidence for baseline depression severity predicting cognitive change in longer studies. Significant results indicated that greater severity was associated with worsening cognitive change, i.e. less improvement, quicker decline or no significant improvement. As the studies were older adults, this may indicate progression of degenerative disease or vascular disease.^42^

#### Diagnosis

##### Adult samples

Three shorter studies (total *n* = 516) investigated diagnosis in relation to cognitive change, one of which found a significant result.

In 353 in-patients, prescribed antidepressant medications as appropriate by their psychiatrist, Lin et al^18^ investigated the effect of depressive subtypes on cognitive change.^43^ A significant relationship was found for psychomotor speed. Those with melancholic depression showed a greater increase in psychomotor speed (Trail Making Test A) over time than those with atypical or undifferentiated subtypes. There was no significant effect of subtype on the remaining five domains of cognitive function.

Miskowiak et al^22,23^ (*n* = 84) examined diagnosis of unipolar or bipolar, and found no effect of diagnosis on improvement in cognitive function.

#### Age at onset

##### Older adult samples

Two shorter studies (total *n =* 239) of older adult samples investigated age at depression onset as a predictor of cognitive change. One study (*n* = 166) of the three reported a significant relationship across their 12-week, open-label trial. Later age at onset was correlated with less improvement in executive functioning (*r* = −0.24, *P* < 0.005).^33^ No relationship was found between age at onset and cognitive change in the areas of working memory, language function, psychomotor speed or episodic memory.^33^ In the remaining study, Nebes et al^30^ (*n* = 73) found no significant relationship between age at depression onset and cognitive change.

##### Longer follow-up studies

Olaya et al (*n* = 291)^39^ investigated age at onset in their 3-year, naturalistic follow-up study of older adults, with non-significant results.

##### Age at onset as a predictor of cognitive change

Late-onset depression with a first episode after around the age of 65 years is associated with greater cognitive impairment and brain changes (vascular changes and hippocampal size^44,45^), and might therefore predict less propensity for improvement in cognitive function during treatment.

The non-significant results from Nebes et al^30^ and Olaya et al^39^ were surprising, as later age at onset of depression in elderly patients tends to be associated with greater cognitive dysfunction.^46^ Indeed, Nebes et al did find significant differences in performance between earlier- and later-onset groups, with later-onset patients performing worse on two of the cognitive tests.^30^ The use of self-report may have affected results of Olaya et al,^39^ as well as their lack of consideration of dementia or mild cognitive impairment, which could have been screened for with a short questionnaire such as the MMSE or the Dementia Rating Scale. Further research could help clarify age at onset as a predictor of cognitive change.

#### Other illness variables

##### Adult samples

Miskowiak et al^23^ (*n* = 79) showed that longer duration of illness was associated with a significant increase in verbal memory (16%) following erythropoietin infusions when adjusted for in their logistic regression with baseline subjective cognitive difficulties, measured with the Cognitive and Physical Functioning Questionnaire, as the predictor. There was no significant increase when baseline memory dysfunction was the predictor, as measured with the Rey Auditory Verbal Learning Test. Number of mood episodes had no significant association with cognitive change in either analysis.

Lee et al^26^ (*n* = 86) examined three other illness-related factors in relation to cognitive change: sick days in the past week before study entry, functional impairment as measured by the Sheehan Disability Scale (SDS) and quality of life as measured by the EuroQol-5 dimensions questionnaire (EQ-5D). For their subjective measure of prospective memory, a higher score on EQ-5D and fewer sick days were associated with greater cognitive improvement. The SDS did not have any significant correlations with cognitive change.

##### Older adult samples

In their eight-week study (*n* = 311), Raskin et al^31^ investigated number of previous episodes of depression, and number of previous medications received for depression. For their composite score, no significant interaction of treatment and these factors was found.

### Cognitive factors

#### Objective baseline performance

##### Adult samples

Baseline objective cognitive performance as a predictor of cognitive change was investigated by eight papers for seven shorter studies (total *n* = 727); five (total *n* = 318) reported significant results.

Miskowiak et al^23^ examined objective baseline verbal memory dysfunction related to cognitive change in an RCT of erythropoietin infusion for moderately depressed patients with unipolar depression and patients with bipolar disorder in partial remission (*n* = 79). For patients with baseline cognitive dysfunction, when compared with patients with no dysfunction, the odds of clinically relevant verbal memory improvement increased by a factor of 290.6. Ott et al^24^ performed a secondary analysis in the same study. When cognitive dysfunction was defined as a score of >1 s.d. below the norm on more than two of the eight cognitive tests, there was deemed to be a significant relationship, in which baseline cognitive dysfunction increased chances of achieving clinically relevant improvement. Patients with cognitive dysfunction were 9.7 and 9.9 times more likely to achieve this at treatment end (week 9) and at 14 weeks, respectively.

Mikoteit et al (*n* = 25)^21^ examined the relationship between baseline cognitive performance and cognitive change (alertness: reaction time *r* = –0.82, *P* < 0.001; working memory: correct responses *r* = –0.40, *P* < 0.1, reaction time *r* = –0.50, *P* < 0.01, ratio of correct responses/reaction time *r* = –0.20, *P* > 0.1; divided attention: correct responses *r* = 0.29, *P* > 0.1, reaction time *r* = –0.80, *P* < 0.001, ratio of correct responses/reaction time *r* = –0.50, *P* < 0.05). Those who had poorer performance at baseline showed greater cognitive improvement. Nadeau et al (*n* = 48)^19^ reported mixed results. Stronger executive functioning at baseline was associated with greater gains in executive functioning. For language, visuospatial function, verbal memory and attention, there was no significant relationship between the baseline score and change within any domain. Barch et al^33^ similarly had mixed results. They found that poorer performance at baseline, as measured by the MMSE, predicted less improvement for working memory; however, they found no significant relationship for executive function, processing speed, episodic memory or language function.^33^

The three remaining studies found no significant relationship between baseline cognitive performance and cognitive change with treatment (total *n* = 409).^16,17,27^

##### Older adult samples

Two shorter studies investigated baseline cognitive functioning (*n* = 517) and found no evidence to suggest baseline cognitive functioning affected cognitive change over their 12-week antidepressant trials.^29,30^

#### Subjective baseline performance

##### Adult samples

Two papers investigated subjective cognitive function at baseline in relation to cognitive change in one sample of patients with current unipolar depression or bipolar disorder in partial remission (*n* = 79).^23,24^ Miskowiak et al^23^ examined subjective cognitive difficulties as measured by the Massachusetts General Hospital Cognitive and Physical Functioning Questionnaire (MGH-CPFQ). Consistent with their own, and Ott et al's objective verbal memory findings above, a significant relationship was reported between subjective baseline dysfunction and objective cognitive outcomes. Odds of the patient having clinically relevant memory improvement increased if they had subjective cognitive complaints at baseline. However, subjective cognitive complaints measured with the MGH-CPFQ, showed no significant relationship between baseline subjective cognitive complaints and change in subjective cognitive function.^24^

##### Baseline cognitive performance as a predictor of cognitive change

Three studies showed that reduced cognitive performance at baseline predicted greater improvement. Baseline objective cognitive performance therefore appears to be one of most consistent predictors of cognitive change. The obvious reason for this is that patients with impaired cognitive performance at baseline are more likely to change. This is likely not only for obvious clinical reasons but also statistically it is more likely improvement can be shown if there are greater deficits. One study conversely found that lower baseline performance predicted less improvement,^33^ although this was a significant result for only one of five domains investigated, making it more likely that this is a chance finding. Additionally, just one study found better baseline cognitive performance was associated with greater cognitive gains, a result they attributed to the phenomenon of those with superior cognitive function being better able to build on this given recovery from depression.^19^ Again, despite examining five domains, only executive functioning showed a significant result.

#### Other biological factors

##### Older adult samples

Barch et al^33^ found that more severe hyperintensities correlated with less improvement in psychomotor speed (*r* = −0.16, *P* < 0.05) in a 12-week trial (*n* = 166). No significant relationship was found for working memory, executive function, language function or episodic memory. Higher vascular risk score correlated with less improvement for working memory (*r* = −0.24, *P* < 0.005) and executive function (*r* = 0.17, *P* < 0.05), No significant relationship was found for language function, psychomotor speed or episodic memory.

One shorter study (*n* = 17) investigated grey matter volumes in relation to cognitive change.^34^ Marano et al^34^ conducted a 12-week trial of citalopram in out-patients with late-life depression, aged over 55 years old. Larger grey matter volumes were associated with greater cognitive improvement, depending on the test and brain area. For verbal learning and memory and letter fluency, greater improvement was associated with larger grey matter volumes in primarily frontal areas. There was also an association between greater improvement in verbal learning and memory and smaller grey matter volumes in the bilateral superior frontal gyrus (BA 8), and an association between greater improvement in letter fluency and smaller grey matter volumes in the bilateral praecuneus (BA 7).

In a 12-week, placebo-controlled trial of donzepil in 18 out-patients with depression and cognitive impairment, Pelton et al reported that worse olfactory performance (University of Pennsylvania Smell Identification Test) at baseline significantly correlated with better episodic verbal memory after the trial.^35^

In one shorter study by Raskin et al^31^ (*n* = 311), several comorbid conditions were investigated as predictors of cognitive change. These were anxiety, insomnia, arthritis, diabetes, vascular disease and mild dementia. No significant interaction of treatment with any of these comorbid disorders was found by their composite cognitive score.

##### Biological factors as predictors of cognitive change

There is evidence in older adult samples that a higher vascular risk score, more severe white matter hyperintensities^33^ and lower grey matter volumes^40^ are associated with less cognitive improvement. Both may be indicators of greater risk for cognitive decline, but both studies were short term.

## Discussion

The aim of this systematic review was to determine what baseline factors are related to cognitive change in patients in a current major depressive episode. The review examined shorter and longer periods of follow-up across adult and older adult samples.

### Predictors of cognitive change

Baseline objective cognitive performance appears to be one of most consistent predictors of cognitive change. There is also limited evidence from the studies identified for age, age at depression onset and depression severity as a predictor of cognitive change, as well as some biological factors. No statements can be made in regards to predictors of cognitive change over a longer term for adults because of a lack of identified empirical data.

### Methodological issues

This review highlighted substantial methodological variability across studies, making synthesis of findings into patterns across studies difficult. The main methodological inconsistencies identified are as follows.

#### Healthy controls

Only three studies included a healthy control group. Without a healthy control group, there is no comparison to ascertain how much of the improvement in cognitive function is because of practice effects or, in the case of longer studies, what effects are a natural progression because of aging. The potential for practice effects may be particularly relevant in studies such as Nebes et al,^30^ who tested their participants on five separate occasions over their 12-week trial.

#### Cognitive impairment, measurement and severity

Although studies tended to exclude participants with dementia, this did not always occur. For example, Raskin et al^31^ allowed ‘mild dementia’ in participants, but excluded Alzheimer's disease. This is clearly important since patients with established dementia will likely have different predictors of cognitive decline. Screening for cognitive impairment was also variable, with some older adult studies using a minimum score on the MMSE as part of inclusion criteria^29,31,33,35,38^ and others including a maximum score to rule out serious cognitive impairment.^36,37^ The majority of the adult studies had no criteria for level of cognitive impairment outside of exclusion of intellectual disability or dementia, and just one had a maximum MMSE score for inclusion.^17^

The cognitive domains investigated by each study also varied. Most studies looked at a variety of cognitive domains, such as executive functioning, verbal memory and attention. There was a range of tests used to measure these. One study examined only global cognitive functioning with the MMSE,^32^ which is likely to be less sensitive. This variety in analysing cognitive performance makes it more difficult to compare and summarise findings.

#### Type of intervention

Around half of the studies used antidepressant therapy, with the remaining half delivering a variety of interventions such as physical activity, cognitive training and rTMS. It may be that different interventions produce different results in cognitive changes, and that different factors predict these cognitive changes. Criteria could have been limited to studies using only one type of intervention, for example, antidepressant treatment. However, if restricted to these studies, results are still very variable, and not sufficiently similar to combine into meta-analyses. Furthermore, we aimed to examine what factors predict change in response to a range of treatments and had no *a priori* hypothesis suggesting that there would be particular predictors for particular treatments.

#### Length of study

The length of the study has implications, in that shorter studies may not be long enough to detect cognitive changes and allow for the identification of predictors. Longer studies, on the other hand, allow for more variables to confound results. This was partially mitigated by the separation of shorter studies (<6 months) and longer studies (≥6 months) in assessing the results. The length of the longer studies, for which follow-up went as long as 10 years,^37^ is also of note, having the issue of more potential confounding variables. Participants may take medications or undergo other therapies during the study period, or have multiple episodes of depression.

#### Sample sizes

Many study samples may be underpowered to detect the effects of predictors. Given the complexity of mood disorders, it is likely that there are multiple interacting predictors.

#### Multiple outcome measurements

Caution should be used when interpreting results in which multiple cognitive outcomes are examined but significant results are reported in just one domain, or test. Any significant results are potentially inflated in this situation, as when testing multiple domains there is more likelihood for type one errors. For example, for age as a predictor, Thomas et al found a significant result for category fluency only, from a battery of 14 tests.^25^

### Limitations

There are several limitations of this review. First, it considered English language papers only. Although this is standard practice for an English language-based review, it may mean that some relevant studies have been missed. Additionally, meta-analysis has not been possible given the heterogeneity of outcome measures, treatments and cognitive domains investigated in the studies examined, and in the variety of ways the data has been analysed and presented.

### Future research recommendations

Clearer analysis (e.g. meta-analysis), and the potential identification of patterns in significance would require more homogeneity between studies; for example, by standardisation of follow-up interval, standard measurement of depressive symptoms and standard batteries of cognitive tests. Failing this, greater numbers of studies performing analysis on what predicts cognitive change would allow for grouping of studies (e.g. by cognitive domain or depression severity) and more in-depth analysis. Given that all studies examining treatment efficacy will be collecting some demographic data at a minimum, this increase is very achievable, with data pooled across studies in international collaborations, for example, the International Consortium Investigating Neurocognition in Bipolar Disorder.^47^

### Conclusions and clinical significance

Evidence from the present review for prediction of cognitive change from baseline variables was limited for demographic factors, with some preliminary evidence for severity of depression and severity of cognitive impairment at baseline as predictors of cognitive change. Identification of patterns across studies was difficult because of methodological variability; for example, variable sample sizes, heterogeneity of patients and measures, and differing study procedures. The identification of predictors of cognitive change could be useful in identifying patients likely to have residual cognitive impairment despite receiving usual treatment. This may be a group of patients who, from the outset, should receive additional support, such as detailed cognitive assessment and specific cognitive rehabilitative therapies.

## Data Availability

Data availability is not applicable to this article as no new data were created or analysed in this study.
